# Investigating intracranial tumour growth patterns with multiparametric MRI incorporating Gd‐DTPA and USPIO‐enhanced imaging

**DOI:** 10.1002/nbm.3594

**Published:** 2016-09-27

**Authors:** Jessica K.R. Boult, Marco Borri, Alexa Jury, Sergey Popov, Gary Box, Lara Perryman, Suzanne A. Eccles, Chris Jones, Simon P. Robinson

**Affiliations:** ^1^ Division of Radiotherapy and ImagingThe Institute of Cancer ResearchLondonUK; ^2^ Royal Marsden NHS Foundation TrustSuttonSurreyUK; ^3^ Division of Molecular PathologyThe Institute of Cancer ResearchLondonUK; ^4^ Division of Cancer TherapeuticsThe Institute of Cancer ResearchLondonUK

**Keywords:** breast metastases, gadolinium‐enhanced MRI, high grade glioma, MRI, USPIO

## Abstract

High grade and metastatic brain tumours exhibit considerable spatial variations in proliferation, angiogenesis, invasion, necrosis and oedema. Vascular heterogeneity arising from vascular co‐option in regions of invasive growth (in which the blood–brain barrier remains intact) and neoangiogenesis is a major challenge faced in the assessment of brain tumours by conventional MRI.

A multiparametric MRI approach, incorporating native measurements and both Gd‐DTPA (Magnevist) and ultrasmall superparamagnetic iron oxide (P904)‐enhanced imaging, was used in combination with histogram and unsupervised cluster analysis using a *k*‐means algorithm to examine the spatial distribution of vascular parameters, water diffusion characteristics and invasion in intracranially propagated rat RG2 gliomas and human MDA‐MB‐231 LM2–4 breast adenocarcinomas in mice.

Both tumour models presented with higher Δ*R*_1_ (the change in transverse relaxation rate *R*_1_ induced by Gd‐DTPA), fractional blood volume (fBV) and apparent diffusion coefficient than uninvolved regions of the brain. MDA‐MB‐231 LM2–4 tumours were less densely cellular than RG2 tumours and exhibited substantial local invasion, associated with oedema, whereas invasion in RG2 tumours was minimal. These additional features were reflected in the more heterogeneous appearance of MDA‐MB‐231 LM2–4 tumours on *T*_2_‐weighted images and maps of functional MRI parameters.

Unsupervised cluster analysis separated subregions with distinct functional properties; areas with a low fBV and relatively impermeable blood vessels (low Δ*R*_1_) were predominantly located at the tumour margins, regions of MDA‐MB‐231 LM2–4 tumours with relatively high levels of water diffusion and low vascular permeability and/or fBV corresponded to histologically identified regions of invasion and oedema, and areas of mismatch between vascular permeability and blood volume were identified.

We demonstrate that dual contrast MRI and evaluation of tissue diffusion properties, coupled with cluster analysis, allows for the assessment of heterogeneity within invasive brain tumours and the designation of functionally diverse subregions that may provide more informative predictive biomarkers.

**Abbreviations**
2D: two dimensional
3D: three dimensional
ADC: apparent diffusion coefficient
ARRIVE: animal research: reporting in vivo experiments
BBB: blood–brain barrier
DW: diffusion weighted
EPI‐DWI: echo‐planar DW imaging
fBV: fractional blood volume
GBM: glioblastoma
H&E: haematoxylin and eosin
IR‐trueFISP: inversion recovery true fast imaging with steady‐state precession
MGE: multi‐echo gradient‐recalled echo
ROI: region of interest
s.e.m: standard error of the mean
SNR: signal‐to‐noise ratio
USPIO: ultrasmall superparamagnetic iron oxide

## 1 INTRODUCTION

The effective treatment of brain tumours is an unmet and urgent clinical need. Five year survival for patients with grade IV glioma (glioblastoma, GBM) is only 5%.^1^ While patients with low grade gliomas have a better prognosis, there is, as yet, no assured cure using conventional therapies. Patients with brain metastases, which affect approximately 8–10% of cancer patients and approximately 30% of patients with breast cancer, have a similarly poor prognosis.^2^, ^3^ High grade and metastatic brain tumours exhibit considerable spatial heterogeneity in gene expression and biochemistry, resulting in regional differences in tumour cell and microvascular proliferation, angiogenesis, necrosis and invasion. Diffuse infiltration of tumour cells in the neuropil, the dense network of interwoven neuronal and glial cell processes, is a characteristic of both low and high grade brain tumours,^4^ and its clinical management is one of the greatest challenges facing neuro‐oncologists and radiologists treating patients with brain tumours.

Imaging biomarkers for the assessment of tumour pathophysiology and response to therapeutics are now widespread in the clinic, and diagnostic imaging is an essential tool in the treatment stratification of patients with brain tumours. MRI enables the visualization of detailed anatomical features with high resolution due to its exquisite soft tissue image contrast,^5^ and is therefore the imaging methodology of choice for defining brain tumour anatomy and delineating tumours. Advanced MRI also provides a means of defining quantitative biomarkers to inform on biologically relevant structure–function relationships in tumours, enabling an understanding of the behaviour and heterogeneous distribution of such associations.^6^ There is a pressing need for refined MRI strategies and quantitative biomarkers to accurately interrogate specific growth patterns associated with infiltrative intracerebral tumours.

Conventional gadolinium‐enhanced MRI, which is used extensively for diagnosis and staging, and in early stage clinical trials in solid tumours, relies upon the hyperpermeable nature of neoangiogenic tumour blood vessels. Infiltrating tumour cells in the brain obtain essential nutrients by co‐opting the existing vasculature, leaving the blood–brain barrier (BBB) intact, and consequently making appropriate delineation of infiltrative brain tumours problematic. Macromolecular ultrasmall superparamagnetic iron oxide (USPIO) particles, which remain within the vasculature over the duration of the MRI timeframe, have been used extensively pre‐clinically to delineate blood vessels and to provide estimates of fractional tumour blood volume and vessel calibre.^7^, ^8^, ^9^ USPIO‐enhanced MRI, alone or in combination with gadolinium‐enhanced MRI, may therefore enable delineation of vessels within glioma regions with an intact BBB within co‐optive, infiltrative areas.

We hypothesized that functional MRI, incorporating assessment of vascular parameters and diffusion characteristics, can report on heterogeneity within infiltrative brain tumours and inform on functionally diverse habitats. We employed a multiparametric MRI approach incorporating native measurements and both Gd‐DTPA‐ and USPIO‐enhanced imaging, and assessed the data using histogram and unsupervised cluster analysis, to examine the spatial distribution of vascular permeability and volume, diffusion characteristics and invasion in RG2 and MDA‐MB‐231 LM2–4 intracranial tumours *in vivo*.

## 2 MATERIALS AND METHODS

### 2.1 Cell culture

RG2 rat glioma cells that stably express pGL4.50[luc2/CMV/hygro] (ATCC: kind gift from Dr D. Crichton, Cancer Research Technology, The Beatson Institute for Cancer Research, Glasgow, UK) and luciferase‐expressing MDA‐MB‐231 LM2–4 highly malignant human triple negative breast adenocarcinoma cells derived from a lung metastasis from an orthotopic MDA‐MB‐231 tumour^10^ (provided by Dr R. Kerbel, University of Toronto, Canada) were maintained in Dulbecco's modified Eagle's medium supplemented with 10% (*v*/v) foetal bovine serum (all Invitrogen, Life Technologies, Paisley, UK). Both cell lines tested negative for mycoplasma infection, and MDA‐MB‐231 LM2–4 cells were authenticated by short tandem repeat (STR) profiling, at the time of tumour propagation.

### 2.2 Animals and tumours

All experiments were performed in accordance with the local ethical review panel, the UK Home Office Animals (Scientific Procedures) Act 1986, the United Kingdom National Cancer Research Institute guidelines for the welfare of animals in cancer research^11^ and the ARRIVE (Animal Research: Reporting *In Vivo* Experiments) guidelines.^12^ RG2 or MDA‐MB‐231 LM2–4 cells (5 × 10^3^) were implanted supratentorially in the brains of 6 week old female athymic (NCr‐*Foxn1* ^*nu*^) mice (Charles River, Margate, UK), as previously described.^13^ A total of 13 RG2 and 14 MDA‐MB‐231 LM2–4 tumour bearing mice were used in this study.

### 2.3 Bioluminescence imaging

Tumour establishment and growth was monitored by bioluminescence imaging using a Xenogen IVIS® 200 system coupled with Living Image software (Caliper Life Sciences, Runcorn, UK). The luciferase substrate luciferin (150 mg kg^−1^, Caliper Life Sciences) was administered intraperitoneally 10 min before imaging. Total photon flux was established for automatically drawn regions of interest (ROIs) at a constant threshold.

### 2.4 MRI

^1^H MRI was performed on a 7 T Bruker horizontal bore microimaging system (Ettlingen, Germany) using a 3 cm birdcage coil and a 2.5 cm × 2.5 cm field of view. Anaesthesia was induced with a 10 ml kg^−1^ intraperitoneal injection of fentanyl citrate (0.315 mg ml^−1^) plus fluanisone (10 mg ml^−1^) (Hypnorm; Janssen Pharmaceutical, High Wycombe, UK), midazolam (5 mg ml^−1^; Hypnovel; Roche, Burgess Hill, UK) and sterile water (1:1:2). Both lateral tail veins were cannulated with a 27G butterfly catheter (Venisystems; Hospira, Royal Leamington Spa, UK) to enable the remote administration of Gd‐DTPA (Magnevist™; Schering, Berlin, Germany; particle size <0.1 nm) and USPIO particles (P904; Guerbet, Villepinte, France; particle size 25–30 nm). Core body temperature was maintained by warm air blown through the magnet bore.

Magnetic field homogeneity was optimized by shimming over the entire brain using an automated shimming routine (FASTMAP). A morphological, 20‐slice, fast, multi‐slice RARE spin‐echo sequence was first used for localization of the tumour and measurement of tumour volume. Next, diffusion‐weighted (DW) images were acquired using an echo‐planar DW (EPI‐DWI) sequence (*T*_R_ = 1500 ms, *T*_E_ = 32 ms, 10 *b*‐values, *b* = 0, 30, 60, 100, 150, 200, 300, 500, 750, 1000s/mm^2^, 4 averages, 3 × 1 mm slices). Subsequently, images were acquired from the same central slice using an inversion recovery true fast imaging with steady‐state precession (IR‐trueFISP) sequence (*T*_E_ = 1.2 ms, *T*_R_ = 2.4 ms, scan *T*_R_ = 10 s, *T*_I_ = 29–1930 ms, 50 inversion times, 8 averages, matrix = 128 × 128, 1 × 1 mm slice, scan duration 10 min 40 s) prior to and 1 min after administration of 0.1 mmol kg^−1^ Gd‐DTPA i.v. (Magnevist). Multi‐echo gradient‐recalled echo (MGE) images (*T*_R_ = 1000 ms, *T*_E_ = 6.2–31.1 ms, 8 echoes, 2 averages, matrix = 256 × 256, 9 × 1 mm slices) were acquired prior to and 2 min after intravenous administration of USPIO particles (150 μmol Fe kg^−1^, 8.38 mg Fe kg^−1^ P904).

### 2.5 MRI data analysis

Parameter estimation was undertaken using a Bayesian maximum *a posteriori* algorithm, which took into account the Rician distribution of noise in magnitude MR data in order to provide unbiased parameter estimates.^14^, ^15^ Estimates of the apparent diffusion coefficient (ADC, ×10^−^^6^ mm^2^ s^−1^) were determined from the EPI‐DWI data. The dual relaxation rate sensitivity of the IR‐trueFISP sequence was utilized to provide estimates of both the native longitudinal and transverse relaxation times, *T*_1_ and *T*_2_ (ms). The change in *R*_1_ (=1/*T*_1_, Δ*R*_1_, ms^−1^) following delivery of Gd‐DTPA was also evaluated. The transverse relaxation rate *R*_2_* (s^−1^) was quantified using the MGE data, and the USPIO‐induced change in *R*_2_* (Δ*R*_2_*) was used to estimate fractional blood volume (fBV, %), using the following equation^7^, ^16^:

$$\mathrm{fBV}=\frac{3}{4\pi }\frac{\Delta R_{2}*}{\gamma \Delta \chi B_{0}}$$

where Δ*χ*, the change in susceptibility induced by USPIO, was taken to equal 0.408 ppm, the Larmor frequency of protons (*γ*) taken to equal 4.26 × 10^7^ s^−1^ T^−1^ and the static magnetic field strength *B*_0_ = 7 T. The value of Δ*χ* is valid for the dose of USPIO contrast agent used *in vivo* in mice.^7^ Pixels corresponding to an fBV exceeding 17% (the limit value for the linearity between Δ*R*_2_* and fBV)^17^ were excluded. All data were fitted on a pixel‐by‐pixel basis using in‐house software (ImageView, developed in IDL, ITT Visual Information Systems, Boulder, CO, USA), and the median value of each parameter determined from an ROI that encompassed the whole tumour. Where possible, ROIs were also drawn over an uninvolved region of the brain in the contralateral hemisphere to the injection of cells, on an imaging slice containing tumour, and the same analyses performed as for tumour ROIs.

### 2.6 Cluster analysis

Unsupervised cluster analysis was performed using in‐house software developed in IDL. The *k*‐means algorithm was employed to partition the ROIs into sub‐regions of similar characteristics, defined in the two‐dimensional (2D) feature space formed by the two parameters Δ*R*_1_ and fBV, Δ*R*_1_ and ADC, or fBV and ADC, and the three‐dimensional (3D) feature space formed by all three parameters.^18^ The optimal number of clusters for each dataset was determined using the cluster validation method described by Sugar and James.^19^

### 2.7 Histological analysis

Where possible, tumour bearing mice were administered with 15 mg kg^−1^ of the perfusion marker Hoechst 33342 (Sigma‐Aldrich, Poole, UK) intravenously through a lateral tail vein with the intact brain being rapidly excised one minute later, snap‐frozen and stored in liquid nitrogen.^20^

Hoechst 33342 fluorescence signals were recorded at 365 nm from frozen whole brain sections (10 μm thick, 3 per tumour) using a motorized scanning stage (Prior Scientific Instruments, Cambridge, UK) attached to a BX51 microscope (Olympus Optical, London, UK), driven by CellP (Soft Imaging System, Münster, Germany). In addition, images of the tumour region only were acquired.

The same sections were then stained with haematoxylin and eosin (H&E), and composite images were acquired using the same microscope system and co‐ordinates under bright field illumination. These images were then used to draw tumour ROIs for analysis of the Hoechst 33342 data. Fluorescent particles were detected above a constant threshold, and the area of the tumour section with Hoechst 33342 fluorescence was determined and expressed as a percentage of the whole tumour area.

The brains from the remaining mice bearing intracranial RG2 or MDA‐MB‐231 LM2–4 tumours (*n* = 5 and *n* = 4, respectively) were formalin fixed and paraffin embedded and 5 μm sections were cut. Sections from two levels through each tumour were stained with H&E, and cellular density assessed by counting the number of nuclei in four square ROIs with a side length of 50 μm, resulting in a total area assessed of 0.01 mm^2^ per field (200× magnification).^21^ Three or more fields per section were assessed.

### 2.8 Statistical analysis

Statistical analysis was performed using GraphPad Prism (GraphPad Software, La Jolla, CA, USA). Results are presented as the mean ± 1 standard error of the mean (s.e.m.); for imaging data the median value is taken for each tumour, and the cohort mean reported. Significance testing used Student's unpaired or paired *t*‐tests, where appropriate, with a 5% confidence level. Frequency histograms were produced (data acquired from all slices in individual animals and from all the animals in the cohort combined), and the degrees of skew and kurtosis were assessed.

## 3 RESULTS

**Figure 1:**
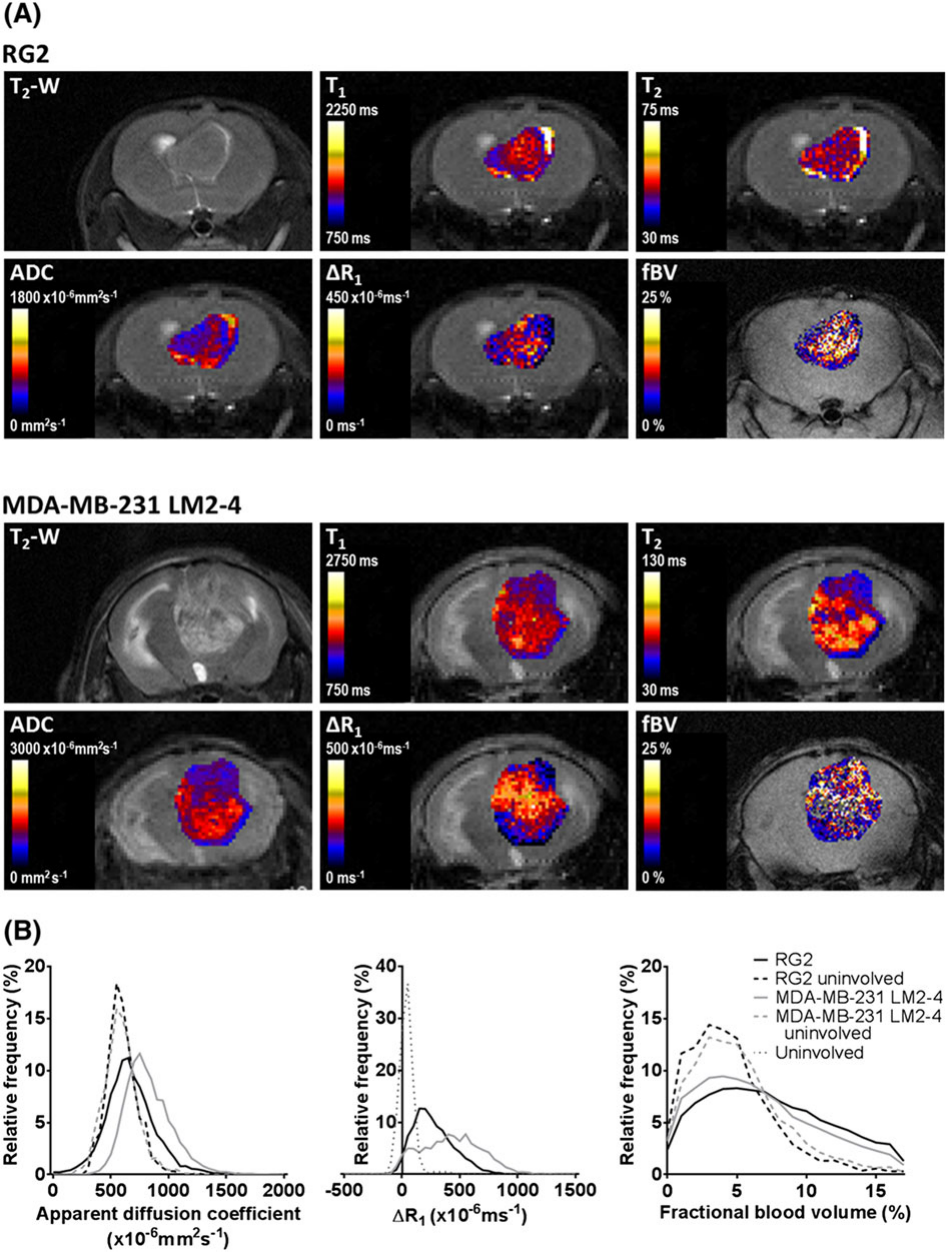
A, *T*_2_‐weighted MRI images and parametric maps of native *T*_1_ and *T*_2_ relaxation times, ADC, the change in relaxation rate *R*_1_ following intravenous administration of Gd‐DTPA (Δ*R*_1_) and fBV, from representative RG2 (upper panels) and MDA‐MB‐231 LM2–4 (lower panels) tumours propagated in the brain. B, Frequency histograms displaying the distribution of ADC, Δ*R*_1_ and fBV in RG2 and MDA‐MB‐231 LM2–4 tumours, and uninvolved brain tissue (data from all evaluated ROIs). Sufficient data could not be acquired from matched uninvolved brain tissue for Δ*R*_1_ analysis; therefore, values from brain tissue in tumour bearing mice where data could be assessed were combined (uninvolved)

A multiparametric MRI protocol, incorporating DW and dual‐contrast‐enhanced MRI, was performed on mice bearing intracranially propagated rat RG2 glioma and human MDA‐MB‐231 LM2–4 breast adenocarcinoma tumours. MRI was carried out when the tumour total photon flux reached a previously established threshold for each model representing a volume of approximately 50 mm^3^ (mean MRI‐derived volume; RG2 47 ± 12 mm^3^, MDA‐MB‐231 LM2–4 53 ± 7 mm^3^, 22 ± 0.2 and 24 ± 2.7 days post injection, respectively). Representative anatomical *T*_2_‐weighted images and parametric maps of native *T*_1_ and *T*_2_, ADC, Δ*R*_1_ and fBV from each tumour model are shown in Figure 1A. The quantitative MRI data for tumour and uninvolved brain ROIs is summarized in Table 1.

**Table 1:** Summary of the quantitative native and contrast‐enhanced MRI biomarkers acquired in intracranially propagated RG2 and MDA‐MB‐231 LM2‐4 tumours and uninvolved regions of tumour bearing brains

	**RG2**	**MDA‐MB‐231 LM2–4**	**RG2 uninvolved (*n* = 6)**	**MDA‐MB‐231 LM2–4 uninvolved (*n* = 7)**	**Uninvolved (*n* = 3)**
***T***_**1**_ **(ms)**	1278 ± 12 (*n* = 5)	1402 ± 18*** (*n* = 7)	—	—	1227 ± 25§§§
***T***_**2**_ **(ms)**	48.3 ± 1.3 (*n* = 5)	58.3 ± 2.5* (*n* = 7)	—	—	41.9 ± 2.1*, §§
**ADC (×10** ^**−**^^**6**^ **mm** ^**2**^ **s** ^**−1**^ **)**	644 ± 16 (*n* = 10)	811 ± 27*** (*n* = 8)	585 ± 9#	576 ± 23####	—
**Δ*R***_**1**_ **(×10** ^**−**^^**6**^ **ms** ^**−1**^ **)**	246 ± 49 (*n* = 5)	420 ± 46* (*n* = 6)	—	—	41 ± 8^*^ §§
**fBV**_**<17**_ **(%)**	6.8 ± 0.2 (*n* = 13)	5.8 ± 0.3** (*n* = 8)	4.0 ± 0.2####	4.4 ± 0.5#	—

Data are mean of median parameter values from each tumour.

* *p* < 0.05,

** *p* < 0.01,

*** *p* < 0.001 versus RG2;

§§ *p* < 0.01,

§§§ *p* < 0.001 versus MDA‐MB‐231 LM2‐4; Student's unpaired *t*‐test;

# *p* < 0.05,

#### *p* < 0.0001 versus matched tumour ROIs, Student's paired *t*‐test.

RG2 tumours revealed a more homogeneous appearance on *T*_2_‐weighted MRI than MDA‐MB‐231 LM2–4 tumours. Whilst both tumour models demonstrated heterogeneity in parametric functional MRI maps, the degree of heterogeneity appeared greater in MDA‐MB‐231 LM2–4 tumours. ADC was higher in both tumours than in uninvolved brain regions of the same animals. Histogram analysis revealed similar shaped distributions of ADC values in both tumour types, but with a shift towards higher values in the MDA‐MB‐231 LM2–4 data, resulting in a significantly higher median ADC (Figure 1B). Intracranial MDA‐MB‐231 LM2–4 tumours also exhibited significantly longer median *T*_1_ and *T*_2_ relaxation times_,_ compared with orthotopic RG2 xenografts.

Administration of Gd‐DTPA increases the relaxation rate *R*_1_ in areas of brain tumours where the vasculature is permeable, for example in areas where the BBB is disrupted. The change in the tumour *R*_1_ (Δ*R*_1_) following Gd‐DTPA administration was significantly greater in MDA‐MB‐231 LM2–4 tumours than in RG2 tumours (Table 1). Insufficient ROIs could be drawn in uninvolved regions on the IR‐trueFISP images in order to perform comparisons on paired regions, but Δ*R*_1_ in both tumour types was significantly higher than that determined from uninvolved regions of both RG2 and MDA‐MB‐231 LM2–4 bearing brains. The frequency distribution of the combined uninvolved ROI data shows that Δ*R*_1_ values in these regions were spread over a narrower range at the lower end of the distribution of the tumour data, but there was no significant difference in skew or kurtosis between the individual ROI frequency distributions.

fBV was significantly higher in both tumour models than in uninvolved regions of the brains of the same animals, and was significantly lower in MDA‐MB‐231 LM2–4 tumours than RG2 tumours (Table 1). Frequency distributions revealed a lower occurrence of fBV values below approximately 7% in the tumour regions than the corresponding uninvolved ROIs, a pattern seen to a lower extent between the MDA‐MB‐231 LM2–4 and RG2 tumours. The frequency distributions of ADC and fBV data in the uninvolved regions of mice bearing RG2 and MDA‐MB‐231 LM2–4 tumours were similarly shaped.

**Figure 2:**
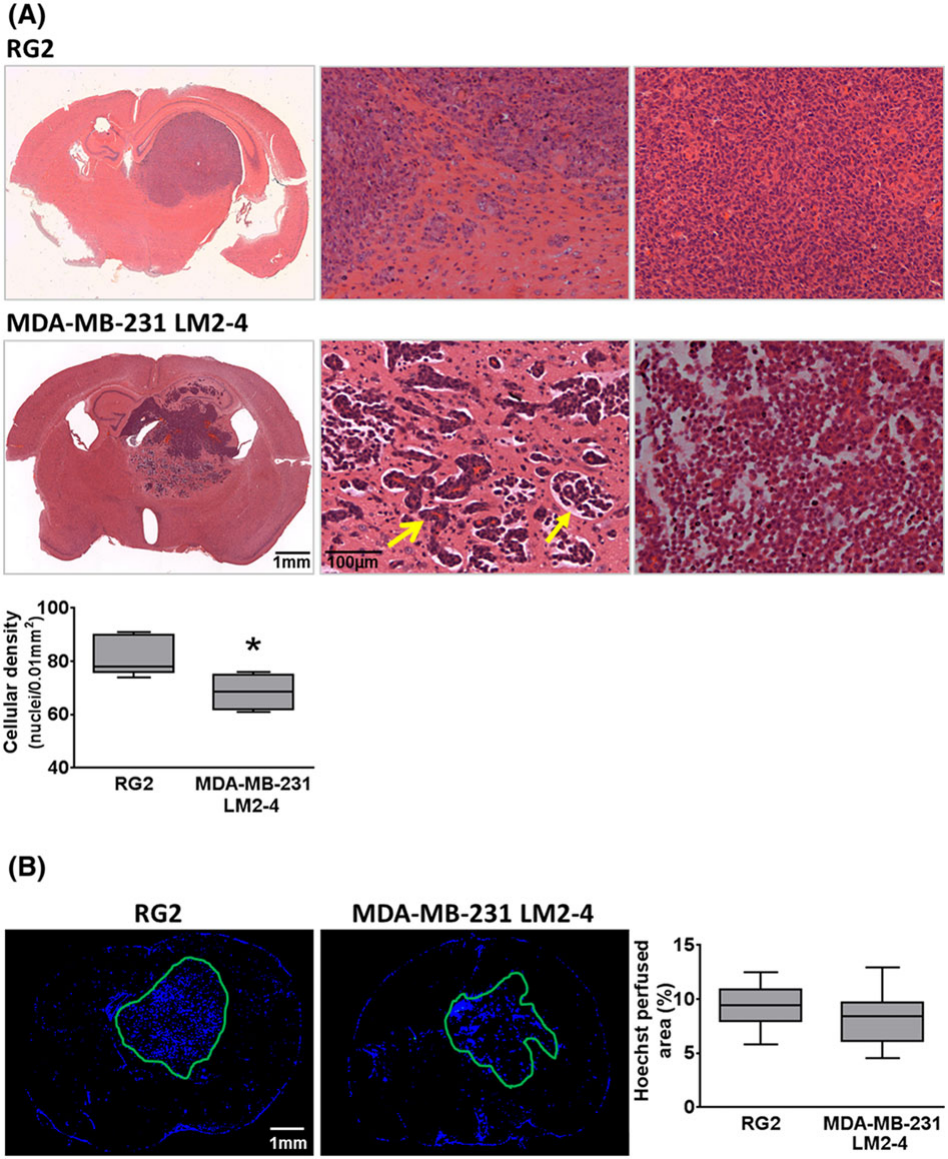
A, H&E staining of RG2 and MDA‐MB‐231 LM2–4; whole brain composite images and 100× images of the tumour periphery (middle panel) showed RG2 tumours as relatively well circumscribed masses with some local invasion at the periphery, and MDA‐MB‐231 LM2–4 tumours as substantially locally invasive (open head arrow shows tumour cells surrounding a blood vessel) with associated oedema (closed head arrow). The right‐hand panel shows cell density at the centre of the tumours. Mean cellular density was assessed in both tumour types; MDA‐MB‐231 LM2–4 tumours were significantly less dense. B, Fluorescence microscopy of Hoechst 33342 uptake in representative RG2 and MDA‐MB‐231 LM2–4 tumour bearing brains revealed no significant difference between the perfused areas in the two tumour types. *p < 0.05, unpaired Student's *t*‐test

H&E staining revealed that tumours derived from MDA‐MB‐231 LM2–4 cells grew as partially well circumscribed masses with substantial local invasion, principally occurring along existing blood vessels (Figure 2A, open head arrow), but also through the brain parenchyma. Tumours derived from RG2 cells grew as well circumscribed masses with limited areas of local invasion typically presenting as small groups of tumour cells or finger‐like projections a small distance from the tumour border (Figure 2A). RG2 tumour cellularity was homogeneous and dense, whereas MDA‐MB‐231 LM2–4 tumours presented with regions of sparse cell density and oedema (Figure 2A, closed head arrow). Quantitative analysis confirmed a significantly lower cellular density in MDA‐MB‐231 LM2–4 tumours (64 ± 5 nuclei/0.01 mm^2^) compared with RG2 tumours (82 ± 3 nuclei/0.01 mm^2^; *p* < 0.05). The Hoechst 33342‐perfused area was assessed and quantified in MDA‐MB‐231 LM2–4 and RG2 tumours (Figure 2B); no significant difference between the tumour types was observed. A positive and statistically significant correlation was determined between Hoechst 33342‐perfused area and MRI‐derived fBV in the RG2 tumours with matched fBV and Hoechst 33342 data (*n* = 4, *r* ^2^ = 0.93, *p* < 0.05) but there was no significant relationship between fBV and Hoechst 33342 perfusion in the MDA‐MB‐231 LM2–4 tumours with matched data (*n* = 5, *r* ^2^ = 0.30, *p* > 0.05).

In order to assess the basis of the heterogeneity observed in intracranial tumours, particularly the more invasive MDA‐MB‐231 LM2–4, unsupervised cluster analysis was used to identify subregions with comparable functional parameters, and therefore similar biological properties, based on Δ*R*_1_, fBV and ADC. When applied to complete fBV datasets, the method identified an ‘outlier’ cluster of high fBV voxels (Supplementary Figure [Link]a). In order to eliminate this cluster and optimize the utility of the cluster analysis, a threshold was applied to each dataset, defined as the value below which a data point had only a 5% probability of belonging to the ‘outlier’ cluster, yielding thresholds of 21.2% for RG2 and 19.7% for MDA‐MB‐231 LM2–4 tumours. These values are in good agreement with the 17% threshold previously applied and reported by Troprès et al. 17, based on the observation that there is no longer linearity between Δ*R*_2_* and fBV above this value. Following this, the optimal number of clusters was then established as 4 for the 2D assessment of Δ*R*_1_ and fBV in the RG2 tumours, and 3 for the 2D assessment of all pairs of parameters and the 3D assessment of Δ*R*_1_, fBV and ADC together in MDA‐MB‐231 LM2–4.^19^

**Figure 3:**
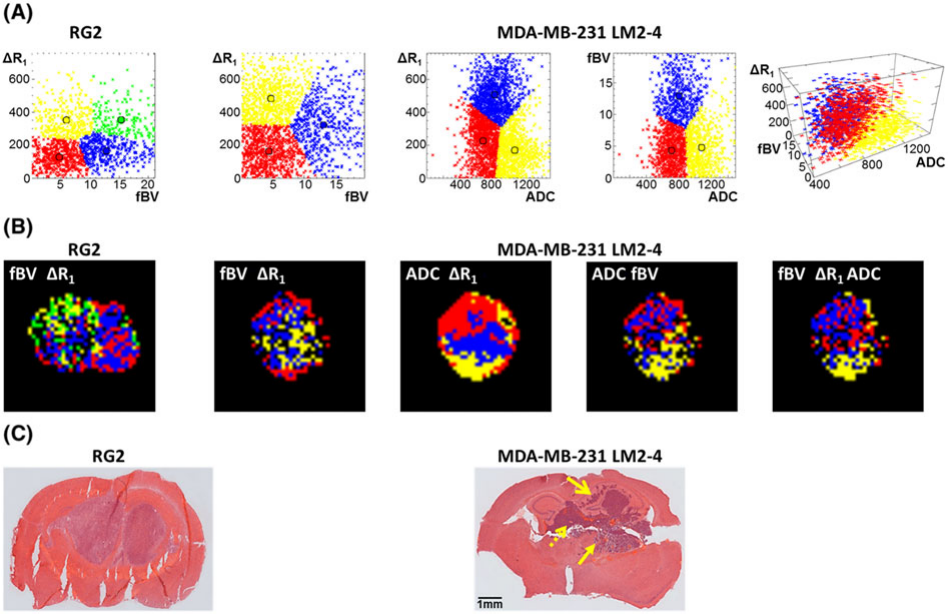
A, Cumulative distribution of voxels in the 2D or 3D space formed by two or three analysed parameters: Δ*R*_1_ and fBV in RG2; Δ*R*_1_ and fBV, Δ*R*_1_ and ADC, fBV and ADC, and all three parameters in MDA‐MB‐231 LM2–4 tumours. Δ*R*_1_ is expressed in units of ×10^−^^6^ ms^−1^, fBV in % and ADC in units of ×10^−^^6^ mm^2^ s^−1^. 2D projections of the clustered 3D voxel distribution are shown in Figure [Link]b. B, Corresponding cluster analysis maps of RG2 and MDA‐MB‐231 LM2–4 tumours; where data thresholding was applied, these values are removed from the maps. Voxels without an evaluable fBV (negative Δ*R*_2_*) are also missing from analyses incorporating fBV. C, H&E staining of the same tumours shows spatial relationships between cluster analysis maps and the tumour physiology. The closed head arrow denotes region of invasion along blood vessels and oedema, the open head arrow denotes invasion without oedema and the dashed arrow denotes main dense tumour mass

Voxel distributions partitioned with the optimal number of clusters, cluster analysis colour maps of a representative tumour slice, and associated H&E images are shown in Figure 3. Figure [Link]b shows 2D projections of the clustered 3D voxel distribution of all three parameters in MDA‐MB‐231 LM2–4, providing a 2D view of the distribution for each parameter pair. In the maps of Δ*R*_1_ and fBV, red voxels, indicative of areas with a low blood volume (low fBV) and relatively impermeable blood vessels (low Δ*R*_1_), were predominantly located at the tumour margins. Yellow voxels originate from subregions with a low blood volume but high vascular permeability. In the MDA‐MB‐231 LM2–4 tumours, all voxels with high fBV clustered together (blue voxels), irrespective of their relative vascular permeability, and were associated with the viable tumour mass. This cluster, driven by relatively high fBV, is also evident in cluster maps combining fBV with ADC and when the contributions from all three functional parameters are combined, although this is coupled with relatively high Δ*R*_1_. In RG2 tumours, however, these high fBV voxels were divided between those with low (blue) and high vascular permeability (green), and were heterogeneously distributed throughout the tumours.

Cluster analysis maps of Δ*R*_1_ and ADC in the MDA‐MB‐231 LM2–4 tumours revealed yellow voxels with relatively high levels of water diffusion and low vascular permeability, which largely corresponded to histologically identified areas of invasion, vascular co‐option and clear evidence of oedema (Figure 3C, closed head arrow). This subregion was also identifiable on cluster analysis maps of ADC and fBV, where high ADC coincides with low fBV and is clearly identifiable on the 3D analysis incorporating all three parameters. Red voxels with low vascular permeability but relatively restricted diffusion were associated with areas of invasion without oedema (Figure 3C, open head arrow), as were voxels ascribed to the red cluster with low fBV and low ADC. Blue voxels with higher Δ*R*_1_ and therefore higher vascular permeability, but with a range of ADC values, corresponded to the main dense cellular mass and largely correspond to the red cluster in the three parameter maps corresponding to relatively low fBV, high ΔR_1_ and low ADC and the yellow cluster in maps incorporating fBV and ΔR_1_.

## 4 DISCUSSION

High grade and metastatic brain tumours exhibit considerable spatial variations in proliferation, cellularity, angiogenesis, invasion, necrosis and oedema. Recent publications have highlighted the potential value of spatial analysis of brain tumours, combining multiple MRI parameters to segment tumour from healthy tissue and to establish regional variations within tumours.^22^, ^23^, ^24^, ^25^ Amongst the greatest challenges facing neuro‐oncologists and radiologists treating patients with brain tumours is diffuse tumour cell infiltration and vascular co‐option. In these regions the BBB remains intact, precluding detection by conventional Gd‐DTPA‐enhanced MRI. We therefore used multiparametric MRI, incorporating both Gd‐DTPA and USPIO particle contrast agents, coupled with histogram and unsupervised cluster analyses, to evaluate the additional information that can be obtained regarding the underlying biology of intracranial tumours when the data acquired from sequences assessing tumour vascular permeability, blood volume and water diffusion are combined.^18^

A key issue at the outset of this study was the application of our multiparametric MRI strategy in intracranial tumour models displaying a clinically relevant invasive phenotype. RG2 rat glioma cells had previously been shown to display an infiltrative growth pattern and very limited extravasation of Gd‐DTPA when propagated intracranially in mice.^26^ In our experience, however, RG2 tumours consistently grew as relatively well circumscribed masses with limited local invasion that demonstrated signal enhancement following administration of Gd‐DTPA, a phenotype consistent with the reported morphology of RG2 tumours grown in syngeneic Fischer 344 rats.^27^, ^28^ Intracranial tumours derived from MDA‐MB‐231 LM2–4 cells, isolated from a lung metastasis following orthotopic implantation of MDA‐MB‐231 cells,^10^ which grow in a partially expansive, partially invasive manner,^13^ were therefore included in this study as a model of invasive tumour growth in the brain. Intracranial injection of parental MDA‐MB‐231 cells has also been used to provide a model of metastatic brain disease.^29^

Interesting differences were apparent in the vascular phenotypes of the two tumour models studied. MDA‐MB‐231 LM2–4 tumours demonstrated higher Δ*R*_1_ than RG2 tumours, indicative of a higher degree of extravasation of contrast agent, hence more permeable vasculature. However, their fBV was lower than that of RG2 tumours, suggesting that, in the simplest terms, high vascular permeability does not necessarily correspond to high vascular density. Cluster analysis of fBV and Δ*R*_1_ allowed for these regions to be mapped spatially; those with low vascular density, but high vascular permeability (Figure 3B, yellow on Δ*R*_1_–fBV map, red on 3D cluster map), are likely indicative of regions of vasculogenesis, where sparse, immature permeable vessels are sprouting to provide a nutritive blood supply to the surrounding tumour cells.^30^ As expected, these regions were typically found within the principal tumour mass. Areas with low vascular volume and low vascular permeability (Figure 3B, red on Δ*R*_1_–fBV map) include those vessels that are not perfused, but may also correspond to areas with a low density of mature, relatively impermeable vessels, likely areas where the native vessels have been co‐opted in regions of invasion.^31^ Indeed, these voxels, particularly in the MDA‐MB‐231 LM2–4 tumours, were predominantly located at the tumour margin, where tumour cell invasion was identified on H&E‐stained sections. Regions with high fBV had either more densely packed or larger calibre vessels, resulting in a relatively larger blood volume. Regions where both parameters were high, which only formed a separate cluster in the RG2 tumours (green), probably correspond to regions with either extensive neovasculature or large distended vessels.^32^

MRI protocols incorporating two vascular contrast agents, either an iron oxide preparation in combination with a gadolinium‐based agent, or sequential injection of low and then high molecular weight gadolinium‐based agents, performed either in the same imaging session or separated by hours, have previously been used to assess tumour vasculature in intracranial tumour models in rats and mice,^33^, ^34^, ^35^, ^36^, ^37^ and for assessing response to anti‐angiogenic treatment in melanoma xenografts in mice.^38^ The key advantage of the imaging strategy used herein is that it enabled the spatial analysis of how the vascular biomarkers assessed by the different contrast agents relate to each other, providing further insight into vascular phenotype and heterogeneity.

Iron oxide‐based contrast agents such as P904 have been used extensively pre‐clinically to assess tumour vascular architecture and function,^39^ but to date no iron oxide preparation has been approved for vascular imaging of patients with cancer. Recent studies have highlighted the off‐label use of ferumoxytol (Feraheme®), an iron replacement therapy for patients with anaemia, as an intravenous MRI contrast agent.^40^ A number of clinical trials are underway, including the assessment of tumour vasculature in combination with gadolinium‐based contrast agents in the same imaging session in adults with primary brain cancer or brain metastases, and in children with brain tumours.^41^ Studies such as these, which incorporate methods similar to those used in this study, may guide the creation of new imaging criteria/biomarkers to evaluate brain tumour progression and pseudo‐progression secondary to radio‐chemotherapy and anti‐angiogenic agents.

Susceptibility contrast MRI exploits negative contrast induced by USPIO particles, which inherently reduce signal‐to‐noise ratios (SNRs). The Bayesian maximum *a posteriori* model used herein provides a thorough treatment of data point estimates of *R*_2_* involving representation of the associated uncertainties. For susceptibility contrast MRI it allows the determination of the significance of differences in *R*_2_* between two or more measurements, and consequently the probability that Δ*R*_2_* is greater than or less than zero on a pixel‐by‐pixel basis *in vivo* can be estimated.^14^ This method represents a more stringent calculation of MRI parameters when SNR is modest or low. Susceptibility artefacts as a result of the air–tissue interface can emerge in MGE images, but any data affected by such artefacts were excluded from analysis in this study.

We were interested to find that, whilst susceptibility contrast MRI‐derived measurements of tumour fBV have previously been validated against uptake of the Hoechst 33342 perfusion marker^42^ and a similar correlation was determined in the RG2 model herein, no such significant relationship was determined in the more invasive MDA‐MB‐231 LM2–4 tumours. We are pursuing further investigations into the pathological assessment of functional vasculature in invasive tumour models in order to understand this relationship more clearly.

In addition to the assessment of tumour vasculature in the brain, the ability to assess cell density in brain malignancies using DW MRI is crucial, particularly where the permeability of the BBB is low. However, care must taken when interpreting ADC measurements, as the relationship between ADC and cell density can be complex, with relatively unrestricted diffusion in areas of oedema and necrosis offset by the reduced ADC in areas of increased cell density. Whilst neither model assessed in this study demonstrated significant necrosis, MDA‐MB‐231 LM2–4 tumours demonstrated evidence of oedema and lower cell density compared to RG2 tumours. This was reflected in higher ADC and native *T*_1_ and *T*_2_ relaxation times, measures of the ratio of bound to free water in a tissue, reflecting increased extracellular space in the tumours.^43^ Histogram analysis of the distribution of ADC values further highlighted differences between tumour types, and compared them with uninvolved regions of the brain. ADC values over 900 × 10^−^^6^ mm^2^ s^−1^ were rare in uninvolved brain regions and uncommon in RG2 tumours, but made up a significant proportion of MDA‐MB‐231 LM2–4 tumour voxels. These high ADC values represent the relatively unrestricted water diffusion in the areas of oedema observed on H&E‐stained MDA‐MB‐231 LM2–4 tumour sections that are absent from RG2 tumours. RG2 tumours were also more cellularly dense, consistent with the higher proportion of pixels with low ADC values.^44^ Interestingly, despite high cellular density and no oedema or necrosis, RG2 tumours had median ADC values and data distributions higher than the uninvolved brain. Assessment of cluster maps of MDA‐MB‐231 LM2–4 tumours incorporating ADC data, alongside H&E staining of the same tumours, revealed clusters of particular note driven by high ADC, denoting relatively unrestricted diffusion. These voxels displayed low Δ*R*_1_ and fBV (low vascular permeability and vascular volume) (Figure 3B, yellow cluster on all 2D and 3D maps including ADC), and corresponded to histologically confirmed regions of invasive tumour growth associated with oedema. A key imaging hallmark of brain tumours is an area of fluid‐attenuated inversion recovery or *T*_2_‐weighted MRI hyperintensity outside the region of contrast enhancement, consisting of a combination of infiltrating tumour cells and oedema.^45^ The routine incorporation of DW imaging to gadolinium‐enhanced imaging protocols in the clinic may thus assist in the more accurate resolution and characterisation of these brain tumour regions.^46^

Multiparametric MRI, focussing particularly on the microvasculature, was used by Coquery et al. to establish MRI‐derived clusters to characterize tumour heterogeneity, and correlate them to pathophysiological features, in rat brain tumour models.^25^

MRI‐based brain tumour segmentation to separate different tumour subcompartments from normal brain structures has been largely performed using supervised learning techniques, which are time consuming and expensive due to the requirement of teaching datasets and labelled images.^47^ Unsupervised methods are now increasingly being developed and have been shown to perform well in comparison with supervised techniques.^24^ Of particular importance is the segmentation of non‐enhancing tumour from healthy tissue to monitor tumour size more accurately over time.^48^

Further refinements in these techniques include 3D histogram analysis of routinely acquired images to identify radiologically defined regional habitat variations in brain tumour data to provide deeper insight into the evolutionary dynamics of brain tumours,^22^ and the incorporation of quantitative functional parameters such as diffusion tensor imaging and dynamic susceptibility contrast MRI into machine learning algorithms, which identified complex and reproducible imaging patterns predictive of overall survival and molecular subtypes in glioblastoma.^23^

In summary, intracranial implantation of MDA‐MB‐231 LM2–4 cells provides a useful model for the development of imaging biomarkers of the vascular and invasive phenotypes of tumours in the brain. We have demonstrated that multiparametric MRI, coupled with unsupervised cluster analysis, allows assessment of the patency of tumour vasculature by simultaneous assessment of both vascular permeability and tumour blood volume, potentially allowing delineation of vasculogenic regions and regions of co‐opted normal brain vasculature. Spatial assessment of tissue diffusion properties in combination with vascular characteristics enabled the relationship between oedema and invasion/vascular co‐option to be evaluated. Development and translation of such techniques, which strengthen the links between *in vivo* imaging biomarkers and the structural and functional properties of tissue, has the potential to provide predictive models to improve brain tumour diagnosis, prognosis and monitoring.

## Supporting information

Supporting info item
