# Supplementary material for: Investigating intracranial tumour growth patterns with multiparametric MRI incorporating Gd‐DTPA and USPIO‐enhanced imaging
Source: NMR Biomed. 2016 Sep 27;29(11):1608–17. doi: 10.1002/nbm.3594 (PMC5082561; doi:10.1002/nbm.3594)
Supplement: Supplementary file 1 — Supporting info item [file NBM-29-1608-s001.docx]

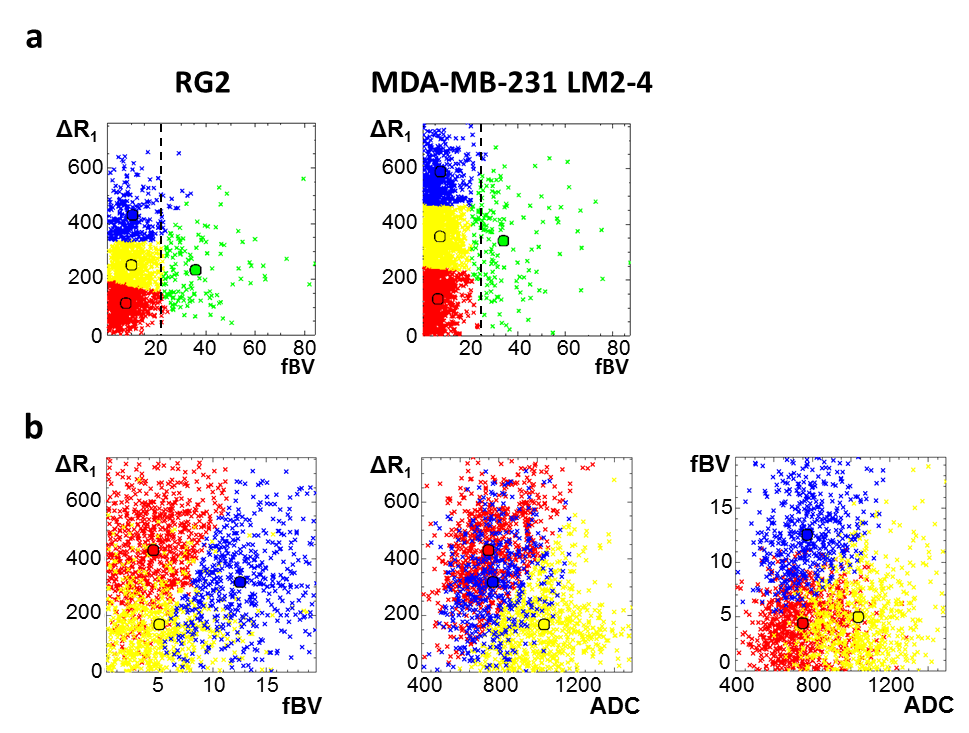


**Supplementary Figure S1**

**a)** Cumulative distribution of voxels in the bi-dimensional space formed by delta R_1_ (ΔR_1,_ x10^-6^ms^-1^) and fractional blood volume (fBV, %) in RG2 and MDA-MB-231 LM2-4 without an upper threshold, showing the cluster distribution dominated by and ‘outlier cluster of high fBV values. Dashed line denotes threshold set as value at which a datapoint has only a 5% probability of belonging to the ‘outlier’ cluster. **b)** 2D projections of the clustered 3D voxel distribution of ΔR1, fBV, and ADC in MDA-MB-231 LM2-4 (Figure 3a), providing a 2D view of the distribution for each parameter pair.
